# Upregulation of circulating microRNA-134 in adult-onset Still’s disease and its use as potential biomarker

**DOI:** 10.1038/s41598-017-04086-w

**Published:** 2017-06-23

**Authors:** Tsai-Ling Liao, Yi-Ming Chen, Chia-Wei Hsieh, Hsin-Hua Chen, Hsiu-Chin Lee, Wei-Ting Hung, Kuo-Tung Tang, Der-Yuan Chen

**Affiliations:** 1 Department of Medical Research, Taichung Veterans General Hospital, Taichung, Taiwan; 2 Ph.D. Program in Translational Medicine and Rong Hsing Research Center for Translational Medicine, National Chung Hsing University, Taichung, Taiwan; 3 Faculty of Medicine, National Yang Ming University, Taipei, Taiwan; 4 Division of Allergy, Immunology and Rheumatology, Taichung Veterans General Hospital, Taichung, Taiwan; 5 Program in Tissue Engineering and Regenerative Medicine, Agricultural Biotechnology Center, National Chung Hsing University, Taichung, Taiwan; 6 Department of Internal Medicine and Medical Education, Taichung Veterans General Hospital, Taichung, Taiwan

## Abstract

Adult-onset Still’s disease (AOSD) is a multi-systemic inflammatory disorder of unknown etiology. To date, no single diagnostic test is available for AOSD. Herein, we investigated the pathogenic role of microRNAs in AOSD. MicroRNA profiles in plasma from AOSD patients and healthy controls were analyzed by microarray analysis, followed by quantitative reverse transcription PCR validation. The biological functions of microRNAs were evaluated using *in vitro* cell-based assay. Among the differentially expressed microRNAs, microRNA-134 (miR-134) expression was positively correlated with AOSD activity scores and significantly decreased after effective treatment. An increased miR-134 level is significantly associated with the activation of Toll-like receptor 3 (TLR3). The reporter assay identified IL-18 binding protein (IL-18BP) as the target of miR-134. A negative correlation between miR-134 expression and IL-18BP mRNA levels were detected in peripheral blood cells following TLR3 ligand treatment. Lower plasma IL-18BP levels and higher IL-18 levels were also observed in active AOSD patients who had higher miR-134 expression than inactive patients. Upregulation of circulating miR-134 was associated with elevated IL-18 levels by targeting IL-18BP in AOSD patients and was positively correlated with disease activity, suggesting its involvement in AOSD pathogenesis. MiR-134 may be a novel activity indicator or potential prognostic biomarker in AOSD.

## Introduction

MicroRNAs (MiRNAs) are short non-coding RNAs composed of approximately 20 to 24 nucleotides that mediate messenger (m)RNA cleavage, translational repression, or mRNA destabilization^1–3^, and currently more than 2,000 human miRNAs are registered (miRBase Release 20.0)^4^. MiRNAs have recently been identified as immune regulators that post-transcriptionally repress target mRNAs expression^5^, and they have diverse functions in immune cell development, particularly the development of Th17 cells^2, 3^. The deregulated expression of miRNAs has been observed in different pathological conditions, including rheumatic and inflammatory diseases^5–8^. Recent studies have revealed that miRNAs are present in a remarkably stable form in plasma and are thought to have potential as clinical biomarkers or therapeutic targets^9–11^.

Adult-onset Still’s disease (AOSD) is a rare inflammatory disease of unknown etiology that usually affects young adults, which is characterized by fever, rash, arthritis, variable multisystemic involvement, and an increase of acute phase reactants^12, 13^. To date, determining predictive factors of outcome and to drawing guidelines for patient management remains difficult^14, 15^. Previous studies, including ours, have demonstrated elevated levels of proinflammatory cytokines including interleukin (IL)-1β, IL-6, IL-18, tumor necrosis factor (TNF)-α, and type 1 T helper (Th1)- or Th17-derived cytokines in AOSD patients^16–21^. Moreover, an increase of spontaneous and IL-18-induced apoptosis plays an important role in AOSD pathogenesis^22^. Curtale *et al*. also demonstrated that miR-146a was involved in T-cell activation and could modulate activation-induced apoptosis^23^. These observations led us to hypothesize that miRNAs may play an important role in AOSD pathogenesis. No data are available, however, concerning the expression of circulating miRNAs in AOSD patients.

In the present study, we investigated differential miRNA expression in plasma from AOSD patients compared with healthy controls using microarray profiling followed by quantitative reverse transcription PCR (QRT-PCR) validation. The associations of candidate miRNAs expression with clinical activity scores or disease outcome were examined in AOSD patients. We used a bioinformatics tool to search for potential targets of candidate miRNAs and subsequently used 3′ untranslated region (UTR) reporter assay for validation. Additionally, we explored the biologic roles of candidate miRNAs in AOSD pathogenesis using an *in vitro* cell-based functional assay.

## Results

### Clinical characteristics of AOSD patients

Among the 12 active untreated AOSD patients in the microarray analysis, common manifestations included spiking fever (9, 75.0%), evanescent rash (8, 66.7%), sore throat (7, 58.3%) and arthritis (5, 41.7%). Lymphadenopathy and hepatosplenomegaly were noted in 4 (33.3%) and 3 (25.0%) patients respectively. There were no significant differences in the age at entry (mean age ± SD, 34.6 ± 12.7 versus 34.7 ± 13.3 years) or in the proportion of females (both were 66.7%) between the AOSD patients and healthy controls (HC).

After initial investigation for miRNAs, all AOSD patients received corticosteroids with/without non-steroidal anti-inflammatory drugs (NSAIDs). The conventional synthetic disease-modifying anti-rheumatic drugs (csDMARDs) used were methotrexate (10 patients), hydroxychloroquine (8 patients), and sulfasalazine (5 patients). During the 2-year follow-up period, 3 patients received therapy with IL-6 receptor inhibitor (tocilizumab).

### Differentially expressed miRNAs using microarray analysis and QRT-PCR

**Figure 1:**
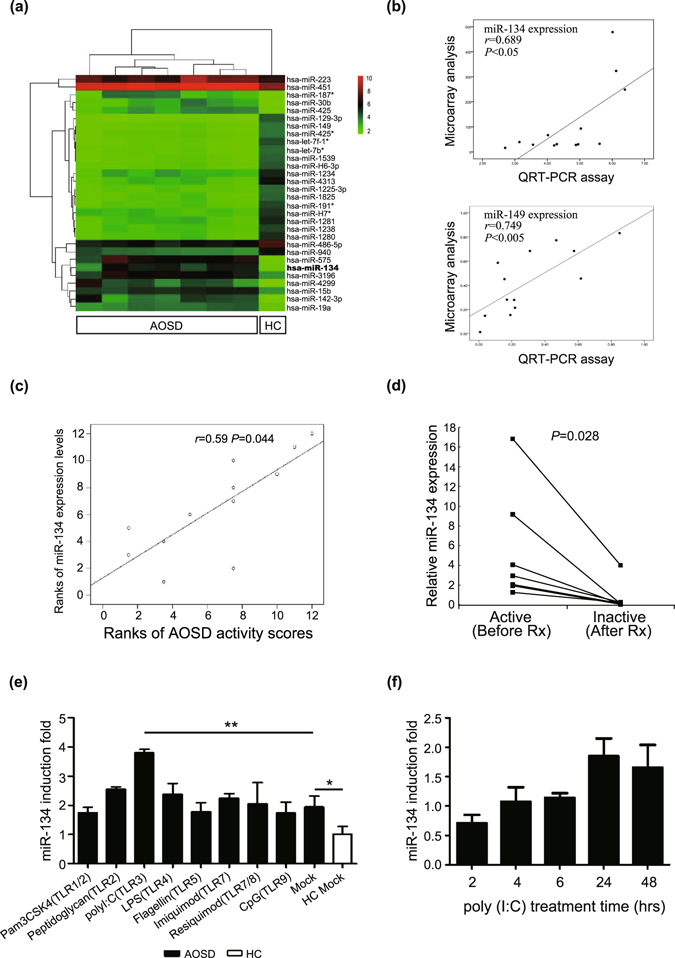
Increased microRNA-134 (miR-134) levels in patients with active adult-onset Still’s disease (AOSD) is associated with disease activity and induced by Toll-like receptor 3 (TLR3) ligand stimulation. (**a**) The differentially expressed microRNAs (miRNAs) in plasma from patients with AOSD and healthy controls (HC) identified using microarray analysis. Hierarchical clustering of miRNA profiles in AOSD patients group and HC group. Relative expression levels of miRNAs are depicted according to a color scale (red represents relative expression greater than the median expression level across all samples and green represents an expression level lower than the median). (**b**) Validation of miRNA microarray with quantitative reverse transcription PCR (QRT-PCR) for the two randomly selected differentially expressed miRNAs in AOSD patients. (**c**) A significant correlation between disease activity and miR-134 expression determined by QRT-PCR assay in AOSD patients. (**d**) Significant decreases in miR-134 expression levels paralleled the clinical remission in AOSD patients after 6 months of therapy. (**e**) Analysis of miR-134 expression in response to a panel of innate immunity Toll-like receptors (TLRs) ligands stimulation. The peripheral blood mononuclear cells (PBMCs) from patients with AOSD were treated with the indicated stimuli for 24 h. MiR-134 expression was analyzed by QRT-PCR and normalized using Rnu6 levels. (f) Kinetics of TLR3 ligand induction of miR-134.

The results of gel electrophoresis confirmed the good quality of RNA isolation in each group as shown in Supplementary Fig. S1. After normalization of the raw data, we observed 28 miRNAs distinctively expressed in plasma from AOSD patients: 12 miRNAs were up-regulated and 16 miRNAs were down-regulated in AOSD patients compared with the HC group (Table 1 and Fig. 1a). Two differentially expressed miRNAs (miR-134 and miR-149) in AOSD patients showed consistent results in both QRT-PCR and microarray analysi**s** (Fig. 1b **)**.

**Table 1:** Differentially expressed miRNAs in plasma from AOSD patients compared with healthy controls, identified by miRNA microarray analysis.

Up-regulated miRNAs	Fold change^§^ (median value)	Down-regulated miRNAs	Fold change^§^ (median value)
hsa-miR-575	18.099	hsa-miR-940	0.169
hsa-miR-4299	14.766	hsa-miR-4313	0.177
hsa-miR-15b	11.140	hsa-miR-1280	0.189
hsa-miR-223	10.089	hsa-miR-1281	0.206
hsa-miR-142-3p	5.860	hsa-miR-486-5p	0.220
hsa-miR-451	5.501	Has-let-7f-1	0.228
hsa-miR-134	4.660	hsa-miR-191	0.232
hsa-miR-187	4.323	hsa-miR-1234	0.243
hsa-miR-19a	3.509	hsa-miR-1825	0.267
hsa-miR-30b	3.507	hsa-miR-129-3p	0.269
hsa-miR-3196	3.491	hsa-miR-1238	0.276
hsa-miR-425	3.322	hsa-miR-1539	0.281
		hsa-miR-149	0.287
		hsa-miR-425	0.294
		hsa-miR-1225-3p	0.295
		hsa-miR-21	0.313

AOSD: adult-onset Still’s disease; miRNA: microRNA.

^§^Fold change: if the number >3.00 or <0.330, the difference is considered significant.

### Correlation between miR-134 expression and activity scores in AOSD patients

Among the differentially expressed miRNAs, the miR-134 expression determined by QRT-PCR assay was positively correlated with the clinical activity scores of AOSD patients (Fig. 1c). However, there were no significant associations of miR-134 expression with clinical manifestations in AOSD patients. Significant decreases in miR-134 expression (mean ± SEM, 5.30 ± 2.19 vs. 0.84 ± 0.56, *P* < 0.05) that paralleled the clinical remission (activity score 5.7 ± 0.5 vs. 2.0 ± 0.4, *P* < 0.05) were observed in AOSD patients after 6-month therapy (Fig. 1d). Although the other differentially expressed miRNA in AOSD patients, miR-149, may be an immune modulator for the TLR/MyD88 signaling pathway in murine macrophages^24^, we did not further investigate its pathogenic role due to the lack of a significant association with AOSD disease activity.

### Association of miR-134 expression and disease outcome in AOSD patients

Defined as described in previous studies^15^, disease outcome including systemic inflammatory pattern and chronic articular pattern was determined for each AOSD patient. Among 23 AOSD patients, 17 (73.9%) had a systemic inflammatory pattern and 6 (26.1%) had chronic articular pattern. As illustrated in Supplementary Fig. S2a, a higher miR-134 expression was observed in AOSD patients with the systemic inflammatory pattern than in those with chronic articular pattern (96.04 ± 35.74 fold vs. 3.04 ± 0.36 fold, *P* = 0.143).

To verify that miR-134 was a potential biomarker for AOSD, we screened circulating miR-134 levels in plasma from patients with AOSD or systemic lupus erythematosus (SLE), which partially shared clinical manifestations with AOSD^25^, as the disease control. The QRT-PCR results (Supplementary Fig. S2b) showed significantly higher miR-134 levels in AOSD patients (n = 30) [active AOSD (n = 12, 128.60 ± 47.72 fold); inactive AOSD (n = 18, 10.00 ± 4.55 fold)] than in SLE patients (n = 22, 2.86 ± 0.53 fold) and healthy controls (n = 22, 5.25 ± 2.26 fold) (both *P* < 0.01). The upregulation of miR-134 was also detected in PBMCs from AOSD patients [active AOSD (n = 6, 147.70 ± 28.25 fold); inactive AOSD (n = 8, 1.91 ± 0.44 fold)], but not in SLE patients (n = 14, 0.16 ± 0.04 fold) or healthy controls (n = 7, 0.53 ± 0.13 fold) (both *P* < 0.0001). (Supplementary Fig. S2c). The upregulation of miR-134 was positively correlated with disease activity (active AOSD vs. inactive AOSD: 147.70 ± 28.25 fold vs. 1.91 ± 0.44 fold, *P* < 0.0001).

### Increased miR-134 levels in PBMCs following TLR3 ligand stimulation

To explore the mechanism related to the upregulation of miR-134 in active AOSD, we further investigated whether innate immunity-associated Toll-like receptors (TLRs) might regulate miR-134 expression. Significantly increased miR-134 levels (3.81 ± 0.09 fold, *P* < 0.01) in PBMCs from AOSD patients after TLR3 ligand polyriboinosinic: polyribocytidylic acid [poly (I:C)] treatment suggest that double stranded RNA (dsRNA) might contribute to miR-134 up-regulation (Fig. 1e). This dynamic result showed that the expression of miR-134 reached a plateau at 24 h after poly (I:C) stimulation (Fig. 1f). To support the findings in AOSD patients, we also examined the miR-134 expression levels in THP-1 cells treated with the different Toll-like receptor agonists including TLR3 ligand, poly (I:C). In the THP-1 cells, significantly increased miR-134 levels were also apparent in cells with poly (I:C) stimulation (1.92 ± 0.11 fold, *P* < 0.01, Supplementary Fig. S3a).

To verify whether poly (I:C)-induced miR-134 upregulation was dependent on TLR3, cells were pre-treated with an inhibitor of endosome acidification (bafilomycin A1), that was known to disrupt TLR3 function^26^. The results showed that bafilomycin A1 had the ability to significantly block poly (I:C)-induced miR-134 expression (Supplementary Fig. S3b). We further confirmed the role of TLR3 in miR-134 expression using TLR3 knockdown assay (Supplementary Fig. S3c). The results showed that there were no significant increases in the miR-134 levels in TLR3 knockdown cells after TLR3 ligand poly (I:C) treatment compared to shLuc knockdown control cells or wild-type cells (Fig. S3c). Altogether, our results showed that TLR3 ligand stimulation induced miR-134 upregulation.

### Cytokine expression levels in miR-134 mimic- or mimic control-expressing cells

**Figure 2:**
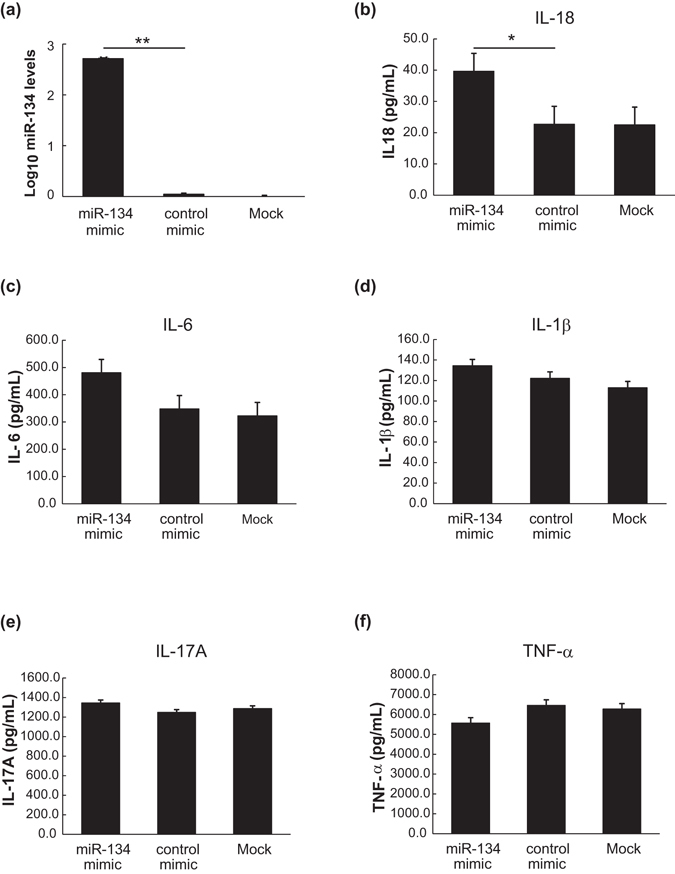
A close link of microRNA-134 (miR-134) expression with levels of IL-18. (**a**) Comparison of miR-134 expression levels in miR-134 mimic-transfected cells, in control-transfected cells, or in non-transfection cells (mock). Comparison of supernatant levels of proinflammatory cytokines including (**b**) IL-18, (**c**) IL-6, (**d**) IL-1β, (**e**) IL-17A, and (**f**) TNF-α released in miR-134 mimic-expressing cells, in mimic control-expressing, and in non-transfection cells (mock) after 24 hours of transfection. Data are presented as mean ± SEM. *P < 0.05, versus mimic control-expressing or in non-transfection cells, determined by the ANOVA test with Scheffe correction.

To explore the biologic role of miR-134, we examined the levels of released proinflammatory cytokines in miR-134 over-expressing cells. As shown in Fig. 2a, markedly higher expression of miR-134 in U937 cells occurred after transfection with miR-134 mimic in contrast to low miR-134 expression in control-transfected cells or non-transfection cells (mock), indicating its effective transfection.

To study the link between miR-134 expressions and AOSD pathogenesis, we examined the levels of proinflammatory cytokines, the reported markers of AOSD^16–21^, in supernatants of miR-134 over-expressing cells. Significantly higher levels of IL-18 were observed in miR-134 over-expressing cells after 24 h of transfection (mean, 39.7 pg/ml) when compared with mimic control-expressing cells (22.7 pg/ml, *P* < 0.05) or non-transfection cells (22.5 pg/ml, *P* < 0.05) (Fig. 2b). However, no significant existed difference in supernatant levels of IL-6, IL-1β, IL-17A, or TNF-α between miR-134 mimic-expressing cells and control-expressing cells or non-transfection cells (Fig. 2c–f).

### MiR-134 targets IL-18BP

**Figure 3:**
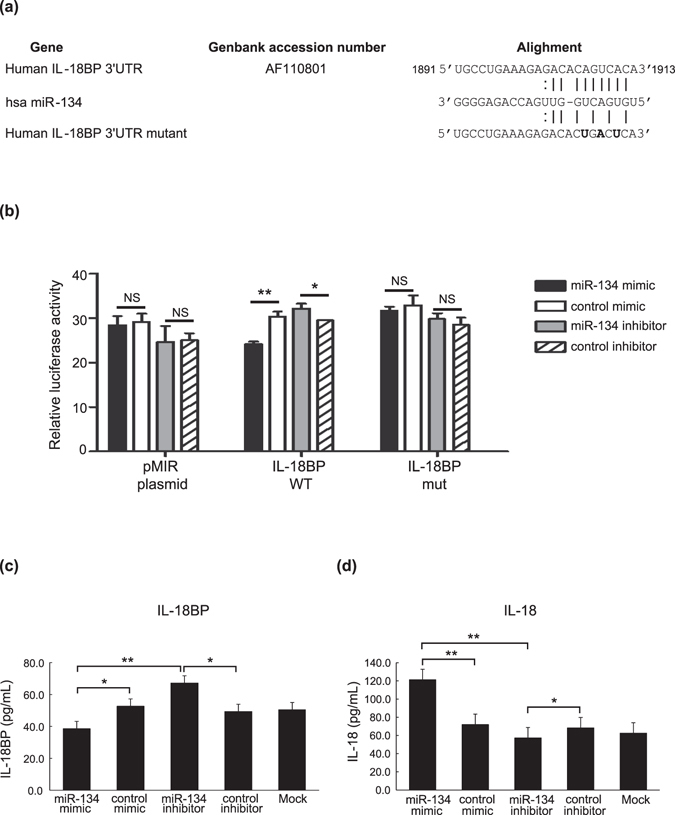
MicroRNA-134 (miR-134) targets IL-18 binding protein (IL-18BP). (**a**) The potential target for miR-134 was IL-18BP. Sequence alignment of miR-134 with reverse complementary IL-18BP (http://www.microrna.org). (**b**) 293 T cells were co-transfected with IL-18BP 3′UTR wild type (WT), IL-18BP 3′UTR mutant or empty pMIR-REPORT vector and pTK-RL plasmid, together with miR-134 mimic/mimic control or miR-134 inhibitor/inhibitor control. After 36 h, luciferase activities were measured and normalized by *Renilla* luciferase activity. Data are presented as mean ± SEM. *P < 0.05, **P < 0.01. NS, non-statistical significant. Comparison of supernatant levels of IL-18BP (**c**) or IL-18 (**d**) released in THP-1 cells transfected with miR-134 mimic, miR-134 inhibitor, mimic/inhibitor control, or non-transfection cells (mock). Data are presented as mean ± SEM. *P < 0.05, **P < 0.01, versus miR-134 inhibitor, mimic/inhibitor control, or non-transfection cells (mock), determined by the Student’s t-test.

Bioinformatics analysis using miRNA target predictions (http://www.microrna.org)^27^ revealed IL-18BP (GenBank: AF110801.1), an intrinsic inhibitor of IL-18, as a potential seed match for miR-134 in its 3′UTR (Fig. 3a).

To validate whether the IL-18BP is the target of miR-134, luciferase reporter plasmid was constructed by cloning the predicted seed sequence in the human IL-18BP 3′UTR into the pMIR-REPORT luciferase vector; the plasmid with the mutation at the pupative binding site was used as a control. Our results showed that miR-134 mimics significantly decreased (*P* < 0.01) while miR-134 inhibitors significantly enhanced the luciferase activity in cells transfected with the IL-18BP 3′UTR plasmid compared to the cells transfected with inhibitor control (*P* < 0.05). No significant change in luciferase activity was observed in cells transfected with the mutant IL-18BP 3′UTR construct or pMIR-REPORT plasmid (Fig. 3b), indicating that IL-18BP is the target of miR-134 and could be regulated negatively by miR-134.

### The expression levels of IL-18BP and IL-18 in miR-134 mimic-, miR-134 inhibitor-, or control-expressing cells

Since IL-18BP is a molecular target of miR-134 and the activated macrophage is the major producer of IL-18^28, 29^, we verified the biologic function of miR-134 in THP-1 cells. As shown in Fig. 3c, significantly lower levels of the released IL-18BP were observed in miR-134 over-expressing cells (mean, 38.6 pg/ml) when compared with miR-134 inhibitor-expressing cells (67.2 pg/ml, *P* < 0.01), mimic control-expressing cells (52.7 pg/ml, *P* < 0.05), or non-transfection (50.4 pg/ml, *P* < 0.05).

Conversely, significantly higher levels of the released IL-18 were observed in miR-134 over-expressing cells (mean, 121.3 pg/ml) when compared with inhibitor-expressing cells (57.3 pg/ml, *P* < 0.01), mimic control-expressing cells (72.0 pg/ml, *P* < 0.01), or non-transfection (62.5 pg/ml, *P* < 0.01) (Fig. 3d).

### TLR3 ligand stimulation induced miR-134 up-regulation, caused reduced IL-18BP levels, and elevated free IL-18 levels in AOSD patients

**Figure 4:**
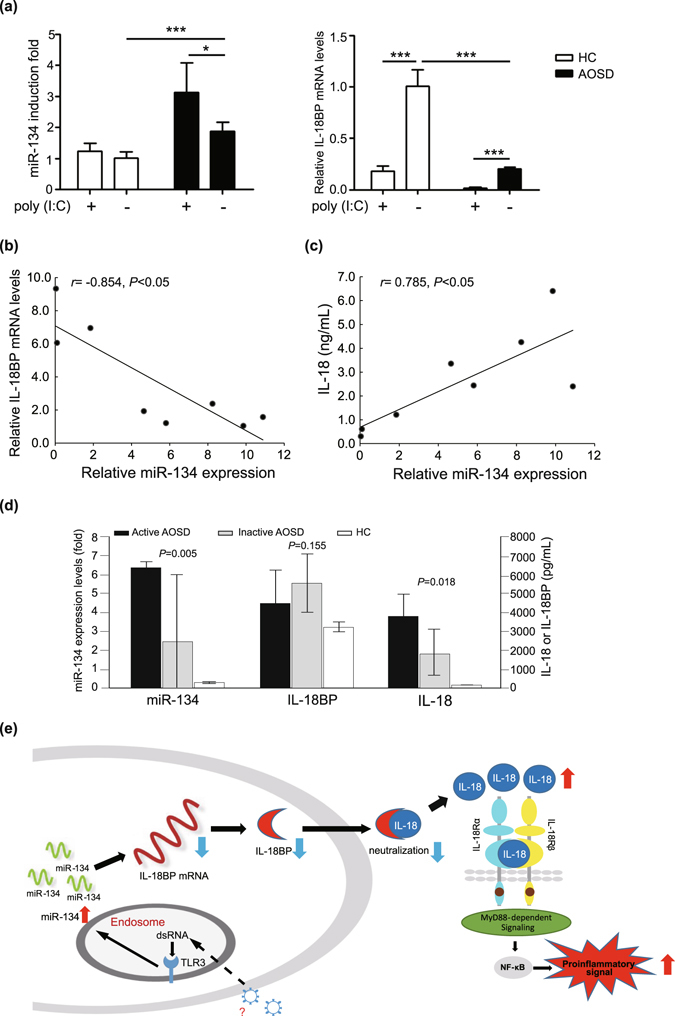
Toll-like receptor 3 (TLR3) ligand-induced miR-134 contributes elevated free interleukin-18 (IL-18) levels by targeting interleukin-18 binding protein (IL-18BP). (**a**) TLR3 ligand poly (I:C) stimulation induced miR-134 up-regulation (left panel), caused significantly reduced IL-18BP mRNA levels (right panel) in PBMCs from AOSD patients or healthy controls (HC). Data are presented as mean ± SEM. *P < 0.05, ***P < 0.005. Correlation between miR-134 expressions and IL-18BP mRNA levels (**b**) or IL-18 levels (**c**) in AOSD patients is determined by Spearman’s correlation test. (**d**) Comparison of expression levels of miR-134, IL-18, and IL-18BP in active AOSD patients, inactive AOSD patients, and HC. Data are presented as mean ± SEM. (**e**) Proposed model for the biologic role of miR-134 in increased level of free IL-18 by targeting IL-18BP in an inflammatory response of AOSD based on the results of this study and previous reports. MyD88, myeloid differentiation primary-response protein 88; NF-ĸB, nuclear factor ĸB.

Due to the significantly increased miR-134 levels in PBMCs from AOSD patients after TLR3 ligand poly (I:C) treatment (Fig. 1e), we further examined the levels of IL-18BP in PBMCs following poly (I:C) treatment. As shown in Fig. 4a, IL-18BP mRNA (right panel) in PBMCs significantly decreased following miR-134 up-regulation (left panel), which is associated with TLR3 ligand stimulation. Additionally, we observed a negative correlation between miR-134 expression and IL-18BP mRNA levels (*r* = −0.854, *P* < 0.05, Fig. 4b) and a positive correlation between miR-134 expression and IL-18 plasma levels in AOSD patients (*r* = 0.785, *P* < 0.05, Fig. 4c).

To verify the results from *in vitro* cell-based assays, we examined the plasma levels of miR-134, IL-18, and IL-18BP in active AOSD patients, inactive AOSD patients, and controls. As shown in Fig. 4d, significantly higher levels of miR-134 and IL-18 were observed in the plasma of active AOSD patients (mean, 6.4-fold, and 3844.0 pg/ml, respectively) compared to inactive AOSD patients (mean, 2.4-fold, *P* < 0.01 and 1863.6 pg/ml, *P* < 0.05, respectively) and controls (mean, 0.3-fold, *P* < 0.001 and 112.0 pg/ml, *P* < 0.001, respectively). Although the finding has no statistical significance, lower plasma levels of IL-18BP were observed in active AOSD patients (mean, 4441.6 pg/ml) compared to inactive AOSD patients (5572.5 pg/ml, *P* = 0.155).

## Discussion

In the present study, we investigated differentially expressed miRNAs in plasma from AOSD patients using a miRNA microarray approach and subsequent QRT-PCR validation. Of the differentially expressed miRNAs, we found a significant association of miR-134 expression levels with disease activity and outcome in AOSD patients. Moreover, a significant decrease in miR-134 levels paralleled disease remission of AOSD, indicating that miR-134 expression may be involved in its pathogenesis and serve as an activity indicator as well as a prognostic biomarker of AOSD. Given that IL-18BP acts as the target of miR-134, identified using 3′UTR reporter assay, we demonstrated significantly lower IL-18BP levels and higher IL-18 levels in miR-134 over-expressing cells and in active AOSD patients who had higher miR-134 expressions compared to inactive AOSD patients. The stimulation from TLR3 ligand contributed to miR-134 up-regulation caused declined IL-18BP expression. Based on these observations, we speculate that miR-134 may enhance free IL-18 levels through targeting IL-18BP, and it may act as a potential biomarker of AOSD disease activity. Confirmation is required from further larger studies.

Toll-like receptors have important roles in recognizing pathogens and in initiating inflammatory responses that subsequently prime specific adaptive immune responses during infection^30^. Additionally, accumulating evidence indicates that TLR activation has an essential role in the pathogenesis of several rheumatic diseases, including rheumatoid arthritis, systemic lupus erythematosus and AOSD^31, 32^. Several studies have reported that TLR activation can modulate miRNA expression in innate immune cells^33^. Our results in the present study show elevated levels of miR-134 following TLR3 activation. TLR3 is primarily responsible for sensing dsRNA, which play a crucial role among viral pathogen-associated molecular patterns. In addition to miR-134, miR-146a and miR-155 have been reportedly induced by TLR3 ligand and regulated proinflammatory cytokine secretion, which were associated with viral infection^34, 35^. In previous studies, the serologic data had suggested that viral infections have a triggering effect in AOSD^36, 37^. Based on our results, we hypothesize that TLR3 might be activated by a viral infection, which then induces miR-134 up-regulation and causes increased circulation of IL-18 in AOSD. Further studies are required to confirm this hypothesis. In addition, our previous study demonstrated that elevated levels of TLR7 signaling molecules and their positive correlation with disease activity in AOSD patients suggest involvement of the TLR7 signaling pathway in the pathogenesis of this disease^32^. No significant association exists, however, between miR-134 levels and TLR7 in the present study, suggesting that miR-134 is specifically up-regulated by TLR3 activation.

MiRNAs have been implicated in important cellular processes, and some research has predicted that one-third of all mRNAs may be regulated by miRNAs^1, 2^. To explore the biologic role of miR-134 and its association with AOSD pathogenesis, we examined the levels of proinflammatory cytokines, the reported markers of AOSD^16–21^, in supernatants of miR-134 over-expressing cells. The significantly higher IL-18 levels in miR-134 over-expressing cells when compared with control cells, without significant difference in the levels of the other cytokines (IL-1β, IL-6, IL-17A, or TNF-α), suggests a close link of miR-134 expression with levels of IL-18, a key cytokine in AOSD pathogenesis^16, 21, 38–42^.

IL-18BP, a protein that efficiently regulates the inflammatory activity of IL-18 as a soluble decoy receptor^16, 40, 41^, has been shown to inhibit IL-18-mediated induction of the nuclear factor ĸB (NF-ĸB) activation and proinflammatory cytokine production by preventing IL-18 binding to its receptor^16, 40, 41^. The low rate of expression of IL-18BP in miR-134 over-expressing cells in our study may lead to an insufficient binding of IL-18. In support of this hypothesis, we demonstrated significantly higher supernatant levels of IL-18 in miR-134 over-expressing cells when compared with control-expressing cells. Moreover, reduced plasma levels of IL-18BP and increased IL-18 levels were observed in our active AOSD patients who had high levels of miR-134 expression, which represents the possibility of an insufficient binding of IL-18 in AOSD. Previous studies similarly found that serum IL-18BP levels were lower and IL-18 levels higher in active AOSD patients than in those with inactive AOSD^43^. Additionally, we revealed a negative correlation between miR-134 expression and IL-18BP mRNA expression (Fig. 4b) and a positive correlation between miR-134 expression and IL-18 expression (Fig. 4c). Recently, Girard *et al*.^42^ demonstrated that free IL-18 levels are specifically elevated in AOSD compared with other inflammatory diseases, suggesting that IL-18 represents a potential target for the treatment of AOSD. Based on our observations and other previous findings^16, 40–45^, we hypothesize that miR-134 induced by TLR3 ligand (dsRNA) stimulation may play a biologic role in the elevation of free IL-18 levels by targeting IL-18BP mRNA and by down-regulating IL-18BP expressions, as shown in the proposed model (Fig. 4e).

Although we arrived at a number of novel findings in this pilot study, it had some limitations. First, the lack of any significant association of miR-134 expression with clinical features may be due to the small sample size associated with this clinically heterogeneous and rare disease: its prevalence has been estimated to be lower than 1 case per 100,000 people^46^. To evaluate the application of miR-134 as a biomarker for AOSD, we compared the expression of miR-134 in patients with SLE, which shared partial clinical manifestations with AOSD^25^, as the disease control. Our results showed that increased miR-134 levels were only detected in AOSD patients but not in SLE patients or healthy controls. Moreover, the upregulation of circulating miR-134 levels was positively correlated with AOSD disease activity, suggesting that miR-134 might be an activity indicator of AOSD. In addition, a higher miR-134 expression was observed in our AOSD patients with systemic inflammatory pattern than in those with chronic articular pattern (96.04 ± 35.74 fold vs. 3.04 ± 0.36 fold), but there was no statistical significance (*P* = 0.143). We thought the decrease of statistical power might be associated with small case numbers of chronic articular AOSD in this study and the confirmation is required from further larger studies. However, our results were consistent with the findings of previous report indicating higher levels of serum IL-18 in AOSD patients with systemic inflammatory pattern compared with those with chronic articular pattern^47^. These observations suggest miR-134 could be a potential biomarker of AOSD. Further large-scale studies are necessary to confirm this hypothesis. Additionally, this study was cross-sectional in design, and, thus, the possibility that miRNA expression changed with therapeutic strategies cannot be excluded. Future studies focusing on miR-134/target relationships *ex vivo* and an in-depth analysis of the pathogenic mechanisms in AOSD are clearly necessary.

To our knowledge, this is the first study to investigate the role of miRNAs in AOSD pathogenesis. We identified 28 differentially expressed miRNAs in plasma from AOSD patients compared to controls. The upregulation of circulating miR-134 was positively correlated with disease activity, indicating its involvement in AOSD pathogenesis, and it may become a novel diagnostic biomarker. Given that IL-18BP is a target of miR-134; our results from the cell-based functional assay suggest that miR-134 may contribute to AOSD pathogenesis through the downregulation of IL-18BP expression and the subsequent elevation of free IL-18 levels. Additionally, TLR3 activation could induce miR-134 upregulation, which is involved in AOSD pathogenesis. Therefore, the blockades of TLR3 or miR-134 inhibitor may provide potential targets for future therapy in AOSD. Further studies are required to confirm and extend the current findings.

## Methods

### Subjects

In the first stage of microarray analysis, 12 consecutive patients with active untreated AOSD who fulfilled the Yamaguchi criteria^48^ were enrolled, excluding those with infections, malignancies, or other rheumatic diseases. The disease activity for each AOSD patient was assessed using a modified Pouchot score^49^. Three age-matched healthy adults without any rheumatic disease were included as healthy controls.

For the replication analysis, we enrolled another 18 AOSD patients to conduct real-time QRT-PCR validation of the differentially expressed miRNAs derived from the microarray analysis. Additionally, we enrolled 22 patients who fulfilled the 1997 revised criteria of the American College of Rheumatology (ACR) for SLE^50^, which shared partial clinical manifestations with AOSD, as the disease control. This study was conducted in compliance with the Declaration of Helsinki and has been approved by the Institutional Review Board of TCVGH (CF11224). The methods were carried out in accordance with the approved guidelines and written consent from all participants was obtained.

### Cell Culture

The peripheral blood mononuclear cells (PBMCs) were immediately isolated from venous blood using Ficoll-Paque^TM^ PLUS (GE Healthcare Biosciences AB, Uppsala, Sweden) density gradient centrifugation. The PBMCs and human monocytic cell lines [U937 (ATCC CRL1593; American Type Culture Collection, Rockville, Md.) or THP-1 cells (ATCC TIB-202)] were grown in RPMI medium 1640 (Gibco, Thermo Fisher Scientific, USA) supplemented with 10% fetal bovine serum (FBS), 1x nonessential amino acids, 100 units/ml penicillin, and 100 units/ml streptomycin in an incubator containing 5% CO_2_ at 37 °C. To readily induce differentiation into macrophages, U937 or THP-1 cells (1 × 10^6^ cells/mL) were grown in media and treated with 10 ng/ml phorbol myristate acetate (PMA) (Sigma, USA) overnight. 293 T cells were cultured in Dulbecco’s modified Eagle’s medium (Gibco, Thermo Fisher Scientific, USA) supplemented with 10% FBS and incubated at 37 °C with 5% CO_2_.

### TLR ligands stimulation

To analyze the expression of miR-134 in response to innate immunity ligands, 5 × 10^5^ cells were treated with the following stimuli for 24 h: Pam3CSK4 (TLR1 and TLR2 ligand, 100 ng/ml), peptidoglycan (TLR2 ligand, 10 μg/ml), poly (I:C) (TLR3 ligand, 50 μg/ml), lipopolysaccharides (LPS) from *Escherichia coli* 055:B5 (TLR4 ligand, 100 ng/ml), flagellin (TLR5 ligand, 100 ng/ml), imiquimod (R837, TLR7 ligand, 1 μg/ml), resiquimod (R848, TLR7 and TLR8 ligand, 1 μg/ml) and CpG (TLR9 ligand, 1 μg/ml)^51^. To analyze the effect of TLR3 inhibitor on miR-134 expression, cells were pretreated with 5 nM bafilomycin A1 (Sigma-Aldrich, USA) for 2 h to disrupt TLR3 function before poly (I:C) stimulation. After treatment, the cells were measured using the TaqMan microRNA real-time RT-PCR Assays kit (Applied Biosystems, Thermo Fisher Scientific, USA).

### TLR3 knockdown

The knockdown reagents were purchased from the National RNAi Core Facility (Institute of Molecular Biology/Genomic Research Center, Academia Sinica, Taiwan). The target of lentivirus-based RNA interference (RNAi) for TLR3 was 5′-CCAGTTCAGAAAGAACGGATA-3′ (TRCN0000056849). The control RNAi was shLuc. Cells were seeded at an appropriate density on 24-well (0.5 ml per well) tissue culture plates and incubated overnight. Cells were maintained in medium containing 8 mg/ml polybrene. The RNAi lentivirus was added to cells at an MOI of 5 and incubated overnight. Then the medium was replaced with fresh medium containing puromycin for selection and incubated at 37 °C with 5% CO_2_. The knockdown efficiency of the target cells was validated using QRT-PCR (Applied Biosystems, Thermo Fisher Scientific, USA).

### MicroRNA isolation

Total RNAs were extracted by TRIzol^®^ Reagent (Invitrogen, Thermo Fisher Scientific, USA) and purified by RNeasy MinElute Cleanup kit (QIAGEN, Germany) according to the manufacturer’s instructions. To extract miRNAs from plasma for QRT-PCR validation, synthetic *Caenorhabditis elegans* miRNA (cel-miR-39, Applied Biosystems, Thermo Fisher Scientific, USA) was added and used as the internal control. Purified RNAs were quantified at OD260 and 280 nm using a ND-1000 spectrophotometer (Nanodrop Technology, USA), and isolated miRNAs were qualified by capillary gel electrophoresis using a Bioanalyzer 2100 (Agilent Technology, Palo Alto, CA, USA).

### MicroRNA microarray analysis

MicroRNA microarray analysis was performed with a total of 887 represented miRNAs (Agilent Technologies, Palo Alto, CA, USA) and a slight modification of the technique, which is described elsewhere^52^. Briefly, one hundred nanograms of total RNAs were dephosphorylated and labeled with pCp-Cy3 using Agilent miRNA Complete Labeling and Hyb Kit (Agilent Technologies, USA). Two- fold hybridization buffers (Agilent Technologies, USA) were added to the labeled mixture to a final volume of 45 μl. Scanned images were analyzed using Feature Extraction software version 10.7.3.1 (Agilent Technologies, USA), and data analysis was performed using GeneSpring 7.3.1 (Agilent Technologies, USA). Signal intensities for each spot were calculated by subtracting local background from total intensities. A median value of the four spots for each miRNA was generated. Normalization was performed using a per-chip 75^th^ percentile method that normalizes each chip on its median, allowing comparison among chips. To highlight miRNAs that characterize each group, a per-gene on median normalization was performed.

### Quantitative reverse transcription PCR (QRT-PCR)

MicroRNA expression was measured and quantified using TaqMan MicroRNA Assays kit (Applied Biosystems, Thermo Fisher Scientific, USA) according to the manufacturer’s protocol. QRT-PCR reactions were performed on the StepOnePlus™ Real-Time PCR System (Applied Biosystems, Thermo Fisher Scientific, USA) using a standard protocol. Each sample was run in triplicate. The small nuclear RNA (Rnu6, for cells) or synthetic cel-miR-39 (for plasma) was used as an internal control gene. The fold expression of the target gene relative to the averaged internal control gene in each sample was calculated using the comparative threshold cycle (Ct) method and evaluated by 2^**−**ΔΔCt^, ΔΔCt = Patient (Ct _miRNAs gene_–Ct _Rnu6/cel-miR-39_) – Mean of controls (Ct _miRNAs gene_–Ct _Rnu6/cel-miR-39_).

For mRNA detection, total RNA was subjected to reverse transcription with oligo (dT)_20_ primer to target mRNA by using SuperScript^®^ First-Strand Synthesis System (Invitrogen, Thermo Fisher Scientific, USA) according to the manufacturer’s instructions. Single-stranded cDNA was subjected to QRT-PCR using the TaqMan^®^ Gene Expression Assays kit (Applied Biosystems, Thermo Fisher Scientific, USA) with specific primer and probe sets. Glyceraldehyde 3-phosphate dehydrogenase (GAPDH) gene was used as an internal control.

### Transfection with miRNA mimics and proinflammatory cytokines release assay

Having demonstrated a significant association of miR-134 expression with disease activity of AOSD, the miR-134 mimic, inhibitor, and control were obtained from Ambion (Thermo Fisher Scientific, USA). U937 or THP-1 cells were transfected with miR-134 mimic, inhibitor, and control (50 nM) using Neon^®^ Transfection system (Invitrogen, Thermo Fisher Scientific, USA) according to the manufacturer’s instructions, and then cells were incubated at 37 °C overnight. To verify miR-134 is associated with AOSD pathogenesis, we chose proinflammatory cytokines as disease markers^16–21^ to detect their expression in miR-134 mimic- or inhibitor-expressing cells. After 24 h, cytokine levels in supernatants were determined using ELISA kits for IL-1β (RayBiotech Inc., Norcross, GA, USA), IL-6 (PeproTech Inc., Rocky Hill, NJ, USA), IL-17A (RayBiotech Inc., Norcross, GA, USA), IL-18 (Medical & Biology Laboratories Co, Ltd., Naka-ku, Nagoya, Japan), and TNF-α (R&D Systems, USA) according to the manufacturers’ instructions. The viability of the remaining cells was determined by the MTT Cell Proliferation Assay (Promega, USA).

### 3′UTR luciferase reporter assays

The wild-type human IL-18BP 3′UTR luciferase reporter plasmid was constructed by amplifying the human IL-18BP mRNA 3′UTR (AF110801.1) and cloning it into the pMIR-REPORT Luciferase vector (Ambion, Thermo Fisher Scientific, USA). Constructs with the AGTCAC to TGACTC mutation at the putative binding site was also generated and used as the control. 293 T cells were co-transfected with 80 ng luciferase reporter plasmid, 40 ng thymidine kinase promoter-*Renilla* luciferase reporter plasmid, and the indicated miR-134 mimic/mimic control (30 nM) or miR-134 inhibitor/inhibitor control (50 nM). After 36 h, luciferase activities were measured using the Dual-Glo Luciferase Assay System (Promega) according to the manufacturer’s instructions.

To verify the biologic effect of miR-134 on the expression of IL-18BP, we also detected IL-18BP levels in supernatants from miR-134 mimic- or inhibitor-expressing THP-1 cells using an ELISA kit (R&D Systems, USA) according to the manufacturer’s instructions.

### Statistical analysis

Results are presented as the mean ± standard deviation (SD) or standard error of mean (SEM). The analysis of variance (ANOVA) test or the Student’s t-test was used for between-group comparison of the expressions of candidate miRNAs or cytokines. The correlation coefficient was calculated using Spearman’s correlation test. Wilcoxon signed rank test was employed to compare the expressions of candidate miRNAs during follow-up in AOSD patients. A probability of less than 0.05 was considered significant.

## Electronic supplementary material

Supplementary information
