# Supplementary material for: Upregulation of circulating microRNA-134 in adult-onset Still’s disease and its use as potential biomarker
Source: Sci Rep. 2017 Jun 23;7:4214. doi: 10.1038/s41598-017-04086-w (PMC5482864; doi:10.1038/s41598-017-04086-w)
Supplement: Supplementary file 1 — Supplementary information [file 41598_2017_4086_MOESM1_ESM.pdf]

Supplementary information

Upregulation of circulating microRNA-134 in adult-onset Still's disease and its use as potential biomarker

Tsai-Ling Liao[1,2], Yi-Ming Chen[1,2,3,4,§], Chia-Wei Hsieh[2,4,§], Hsin-Hua Chen[1,2,3,4,§], Hsiu-Chin Lee[1,5], Wei-Ting Hung[3,6], Kuo-Tung Tang[2,4], Der-Yuan Chen[2,3,4,6*]

[1]Department of Medical Research, Taichung Veterans General Hospital, Taichung, Taiwan

[2]Ph.D. Program in Translational Medicine and Rong Hsing Research Center for Translational Medicine, National Chung Hsing University, Taichung, Taiwan

[3]Faculty of Medicine, National Yang Ming University, Taipei, Taiwan

[4]Division of Allergy, Immunology and Rheumatology, Taichung Veterans General Hospital, Taichung, Taiwan

[5]Program in Tissue Engineering and Regenerative Medicine, Agricultural Biotechnology Center, National Chung Hsing University, Taichung, Taiwan

[6]Department of Internal Medicine and Medical Education, Taichung Veterans General Hospital, Taichung, Taiwan

[§]These authors contributed equally to this work.

[*]Address reprint requests and **correspondence** to: Dr. Der-Yuan Chen, Department of Internal Medicine and Medical Education, Taichung Veterans General Hospital, Taichung, Taiwan. No. 1650, Sec. 4, Taiwan Boulevard, Taichung 40705, Taiwan

E-mail: dychen@vghtc.gov.tw

Tel.: +886-4-23592525 ext. 4300 Fax: +886-4-23503285

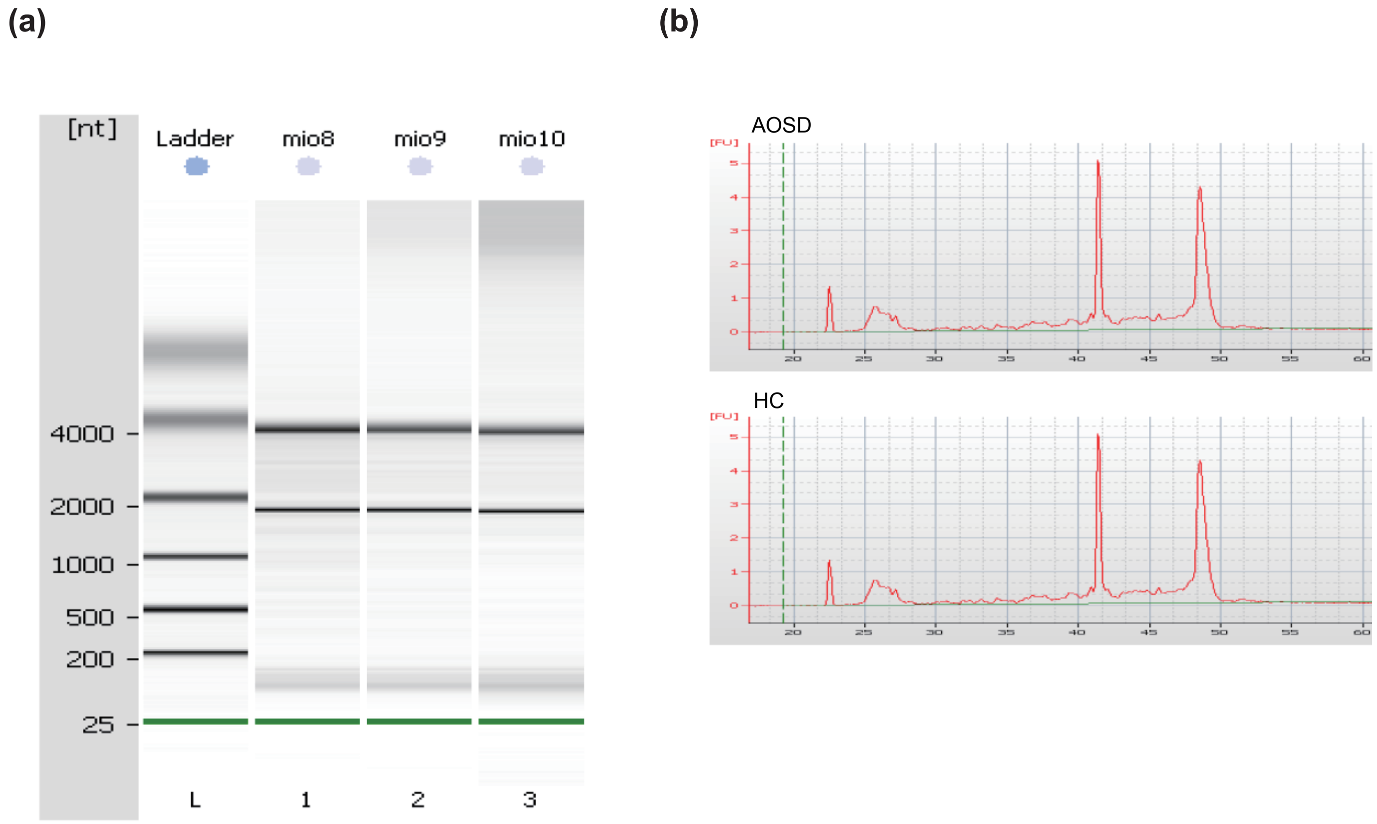

Supplementary Figure S1. (a) Gel electropherograms for the size distribution of enriched small RNAs in plasma from one representative adult-onset Still's disease (AOSD) patient and healthy control (HC). (b) The y-axis represents fluorescence units (FU) and the x-axis runtime in seconds (s).

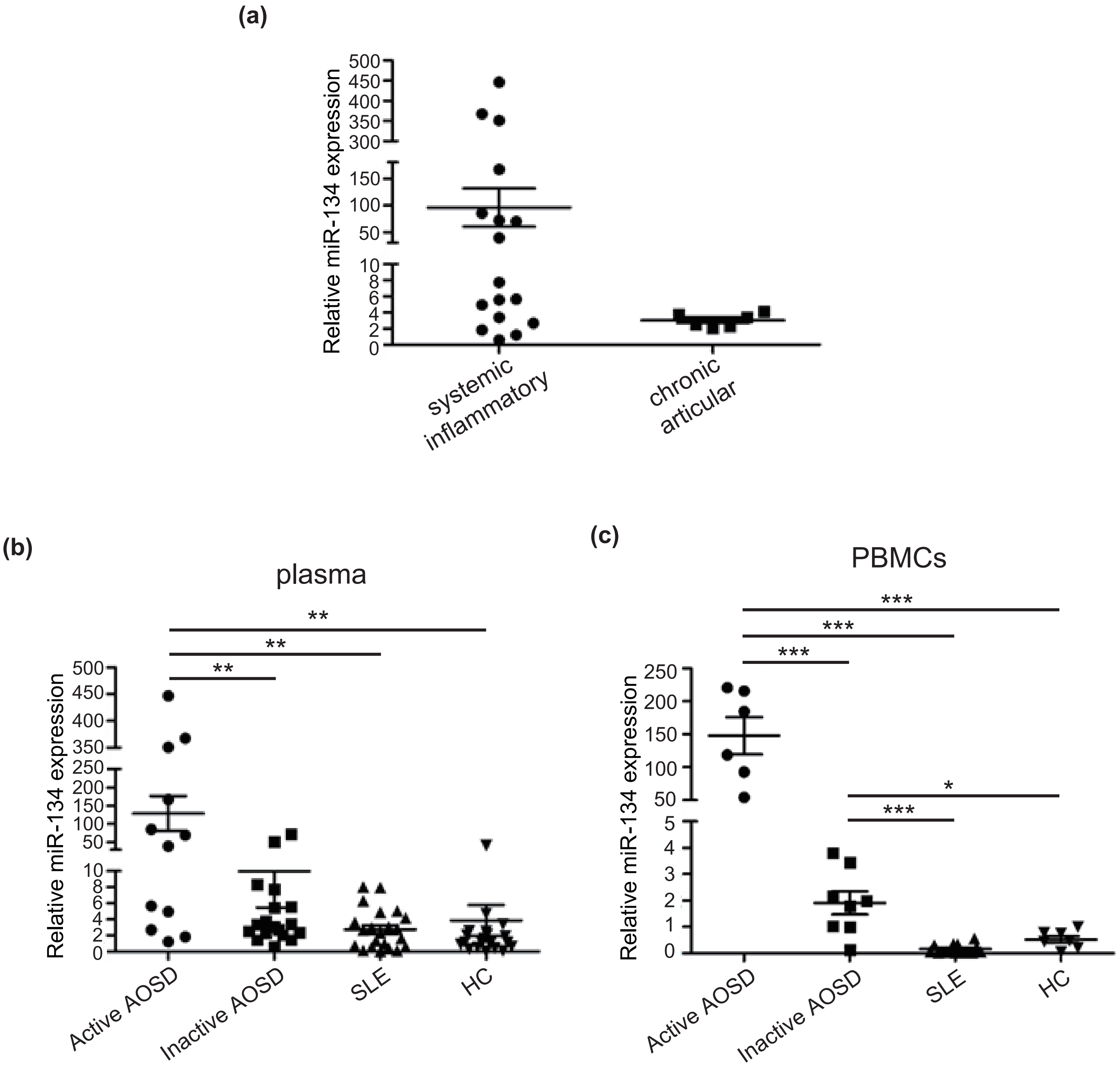

Supplementary Figure S2. Increased circulating microRNA-134 (miR-134) levels in patients with adult-onset Still's disease (AOSD) are associated with disease outcome. (a) The differentially expressed miR-134 levels in plasma from AOSD patients had different disease outcomes. Comparison of circulating miR-134 levels in plasma (b) and peripheral blood mononuclear cells (PBMCs) (c) from patients with active AOSD, inactive AOSD, systemic lupus erythematosus (SLE) and healthy controls (HC). Data are presented as mean±SEM. * *P*<0.05, ** *P*<0.01, *** *P*<0.0001, determined using Student's t-test.

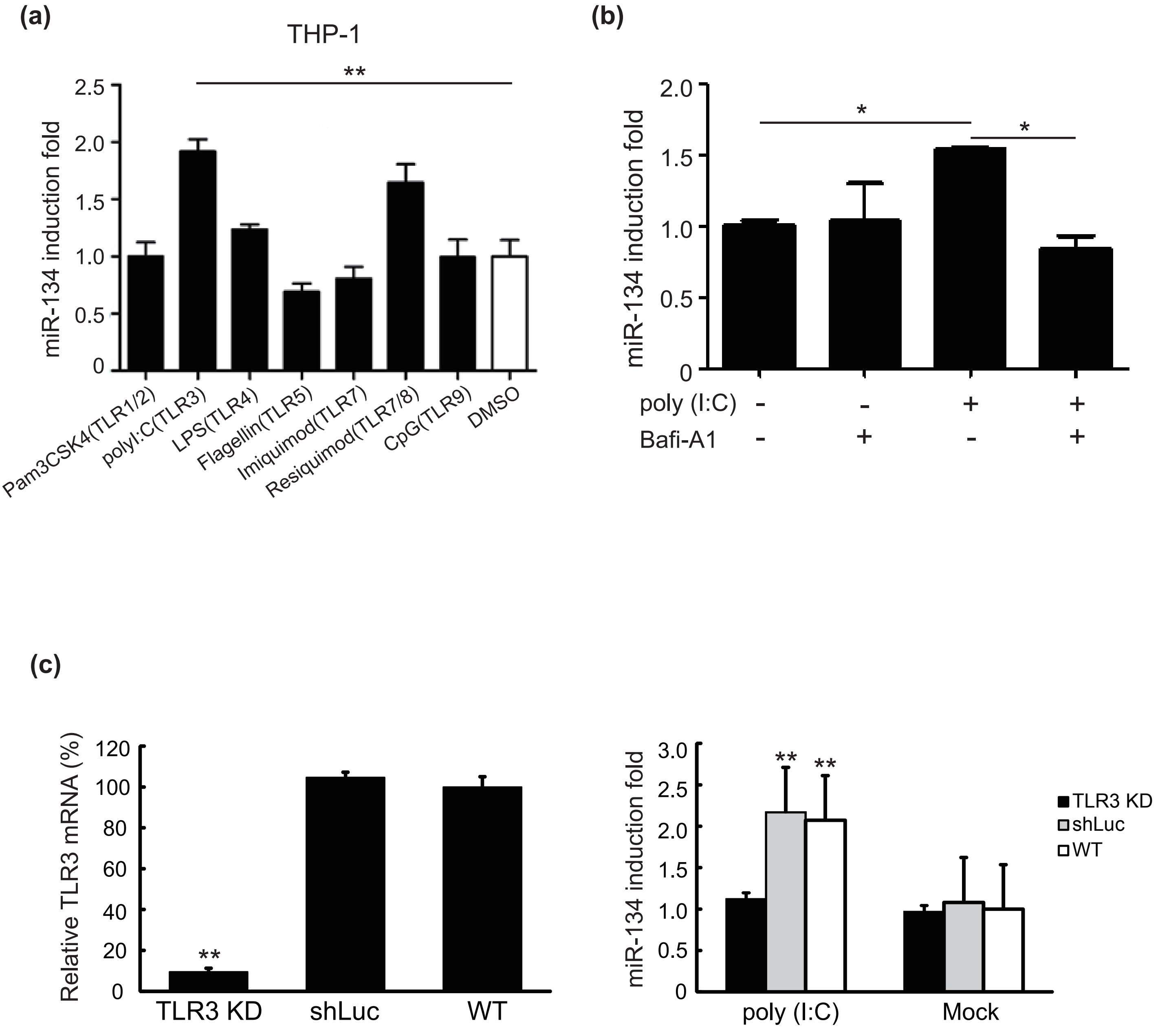

Supplementary Figure S3. Poly (I:C)-induced miR-134 upregulation is dependent on Toll-like receptor 3 (TLR3). (a) Analysis of miR-134 expression in response to a panel of innate immunity-associated TLR ligand stimulation in THP-1 cells. (b) Bafilomycin A1 (Bafi-A1) significantly blocks poly (I:C)-induced miR-134 expression. (c) For TLR3 knockdown validation, TLR3 mRNA expression was examined using real-time PCR (left panel). No significantly increased miR-134 levels in TLR3 knockdown (TLR3 KD) cells with poly (I:C) treatment (right panel). Data are presented as mean±SEM. * *P*<0.05, ** *P*<0.01, determined using Student's t-test.
